# On Obliteration of the Veins, as the Cause of Œdema, or Partial Dropsy, and Particularly in the Inferior Extremities

**Published:** 1831-08

**Authors:** M. Corbin

**Affiliations:** Physician to La Charité.


### OBLITERATION OF THE VEINS.

*On Obliteration of the Veins, as the Cause of Œdema, or Partial Dropsy, and particularly in the inferior Extremities.* By M. CORBIN, D.M.P., Physician to La Charité.*

OBSTRUCTION of the veins has been mentioned by various authorities as the cause of certain partial dropsies, and, among others, by MORGAGNI, CAMPER, HODGSON, TRAVERS, BECLARD, RAIKEM, and BODSON. The interesting article by M. RAYER (Dict. in 21 vols. *Hydropisie*,) and two memoirs by M. BOUILLAUD (Archives, 1823 and 1824,) contain a satisfactory account of all that had been previously done upon this subject. To M. Bouillaud, however, the credit is due of having completely established the fact, that dropsy does sometimes depend upon this cause: he relies solely upon facts, and shews successively obstruction of the abdominal

* Archives Gen., Avril 1831.

vena cava giving rise to œdema of both inferior extremities; of one iliac or femoral vein, to infiltration of one limb; of the vena porta, to ascites; of the superior cava, or of the larger trunks which join it on the right or left side, to infiltration of the whole face and upper extremities, or to one half of the face and one arm. In this theory M. Bouillaud includes passive dropsies alone, and not inflammatory dropsies. Other pathologists, as Meckel, Travers, Chaussier, David Davis, and M. Velpeau, have attributed to a similar cause the acute œdema of recently delivered women.* Several writers have recently endeavoured to confirm and still further to develope certain parts of M. Bouillaud's theory. M. Reynaud has shewn, by additional facts, that obstruction of the vena porta produces ascites, and that it also gives rise to a dilatation of the superficial veins of the abdomen. This latter observation is highly important in a diagnostic point of view.

On the other hand, opponents to this system are not wanting. Hodgson, Travers, and Beclard have recorded some cases which favor the views of M. Bouillaud, and others which are opposed to them. MM. Rayer and Bricheteau (Dict. in 21 vols. *Hydropisie*,) tied the principal venous trunks, but no serous effusion was produced; and Bichat maintained that obstruction of the veins, and even of their principal trunks, did not in general give rise to dropsy.

It is not my intention to enter into the discussion of the general question: I shall endeavour to illustrate one point only; the case in which one inferior extremity alone is infiltrated, or a great deal more infiltrated than the other. Morgagni, Travers, Hodgson, and Bodson have related four cases of this sort, and M. Bouillaud details four others in his first memoir: in all these instances there was an obstruction in the great venous trunks of the affected limb. It may not, however, be useless to add to the number of these cases, as we shall thus be led to draw more positive conclusions. Following the example of M. Bouillaud, I refer only to passive dropsies, and leave entirely out of the question what others have written respecting that painful species of œdema which occurs in puerperal women.

Case I. Joseph Lalique, æt. thirty-one, was admitted into the Charité, April 20th, 1829: he had for many years been troubled

* Dr. Robert Lee has greatly contributed to prove this pathological doctrine: he has some very interesting preparations of obstruction in the iliac veins, which were taken from patients who had laboured under phlegmasia dolens; the "acute œdema" of M. Corbin.—Editor.

with cough and abundant expectoration; he had night sweats and diarrhœa, and was reduced to the last stage of marasmus. Upon examining the chest, tubercular disease, in an advanced stage, was detected, especially of the right side; but the symptom that particularly attracted attention was an œdematous swelling of the left lower extremity, which was nearly one third bigger than the right. The limb was painful and heavy; slight motion of it caused great distress. It was examined with particular attention, as there was no appearance of anasarca in any other part of the body. The vena saphena, and its divisions, appeared healthy, as far as could be determined on account of the swollen state of the limb. In the course of this vein no particular pain was felt, nor was there any redness of the surface in any part of the limb.

The patient died on the 1st May, and his body was examined next day. The state of the lungs was not the particular object of inquiry, but the following appearances were found in the œdematous limb. The vena saphena, and its branches, were empty, healthy, and of a natural size. The external iliac vein, from about an inch before its passage under Poupart's ligament, the whole course of the crural vein, and all its deep-seated divisions, from their origin to their most minute branches, were converted into solid knotty cords. In the interim of these veins there were firm fibrinous clots, of a pale red colour: in some parts the clots were rather soft, and like blood recently coagulated; in others still softer, with the appearance of grumous sanies, of the colour of the lees of red wine: such was, especially, the state of the blood in the iliac, and the commencement of the crural vein. The parietes of these veins were opaque, firm, and elastic, of a dull white colour: in the other limb, no similar appearances existed.

Case II. A young man, named Millot, twenty years of age, was admitted, December 10th, 1828, into the Charité, in a state of great exhaustion: he died on the 18th January. From the symptoms he had previously laboured under, it was presumed he had had a pneumonic affection, combined with ulceration of the intestines. As in the preceding case, the left inferior extremity was œdematous: the limb had swollen suddenly on the 3d or 4th of January: the infiltration was considerable from its first appearance, and continued to increase to such an extent that a very deep pit was made on the surface by pressure. The whole limb was painful: there was no redness of the skin, nor any apparent lesion of the veins, or any other part, which could account for the œdema.

*Dissection.* The left iliac vein, for about two inches above the crural arch, was filled with soft red grumous matter ("bouillie,") in which we thought we could detect a mixture of blood and pus. Above this part the vein was unobstructed, as was also the vena cava, which was examined as far as the diaphragm. Below, and down to the heel, the crural vein, and all its deep-seated ramifications, were filled and clogged up by a firm clot, which, for about five inches from the origin of the crural vein, was detached from the parietes, having a smooth round appearance, like a worm. In the iliac vein, a layer of brown, flocculent, thin fibrine was deposited upon the parietes, and separated them from the pultaceous mass above mentioned: it was easily detached, and raised in long shreds, like those which are found in the interior of aneurisms. The parietes of the iliac and crural veins, and the branches which were thus obstructed, were decidedly thickened and opaque. Around the veins, down the whole of the limb, the cellular tissue was preternaturally firm. At the groin, this hardened state of the cellular tissue was particularly observed: the lymphatic ganglions at this part were also indurated, but not much increased in size. The veins of the other limb were healthy.

In these cases the infiltration existed only on one side, and the vessels of the affected limb were filled with coagulated blood; but in the following instances the facts were different.

Case III. A young Englishman, æt. twenty-three, was admitted, under M. Chomel, for a long-standing pleurisy of the right side, to which had succeeded effusion into the left cavity of the chest. Every means were, in vain, adopted to save him. From the commencement of his illness, the inferior extremities were slightly œdematous. The infiltration was much increased towards the end of the disease, and particularly in the left limb: the tumefaction was indolent, without superficial redness, or any indication of any lesion of the vessels; the inguinal glands were healthy.

*Dissection.* Throughout the whole track of the crural vein, and in most of its branches, and also in the vena saphena and its divisions, were found sanguineous concretions, apparently of recent formation, of a soft consistence, and deep colour: in some points, however, both in the large and small veins, the concretions were less firm, and of a paler colour. In the other limb no appearances of the kind were detected.

Case IV. Levasseur, æt. forty-six, was admitted into the Charité, January 30th, 1829: he died, the 9th of February, of an abscess of the liver, complicated with peritonitis and slight pneumonia. A few days before his death, both inferior extremities became œdematous; the left limb was most swollen. Notwithstanding the difference in the size of the two extremities, it had been presumed that the infiltration in them depended upon effusion within the peritoneum. The vessels of the right inferior extremity were healthy. The termination of the external iliac vein for about an inch above the crural arch, the crural vein, saphena, and all its ramifications on the left side, were converted into large, knotty, and hard cords. The proportional size of the veins may be imagined from that of the saphena, which was nearly the size of the little finger. All these veins were filled with a firm fibrinous coagulum. At the upper part of the vena saphena and crural vein, as well as in the iliac vein, the coagulum was of a brown pultaceous consistence. The parietes of these veins were thickened, and of a dull white colour; the valves retained their delicate structure and transparency.

In the two last cases, the limb, whose vessels were found healthy, had been but slightly infiltrated during life. In the following case, in which there was also a slight œdematous appearance, the vessels were not entirely free from obstruction.

Case V. Victor Damoisel, æt. forty-three, entered the Charité, June 17th, 1829: he had long been in a consumption, and his speedy death was inevitable. The right inferior extremity was very œdematous; the left so slightly as not to be perceived by the eye; the œdema could only be detected by pressure. In about ten days he died.

*Dissection.* The termination of the inferior vena cava, from about an inch above its bifurcation, contained some clots of coagulated blood, of a light violet colour. The common iliac and the external iliac veins of the left side also contained similar coagula, in a greater number, but still they were not sufficiently numerous to fill the vessels, and to offer a complete obstruction to the circulation of the blood. On the right side, the common and external iliacs, and all the superficial and deep-seated veins, from the crural arch to the foot, were filled and distended with coagulated blood, which was of a firm consistence in the smaller branches only. In the large veins, and particularly in the external iliac and origin of the crural, there were merely clots of grumous blood, of different sizes, mingled with reddish-looking sanies, partly floating and partly adherent to the parietes of the vessel. The parietes of the veins were thick, firm, opaque, and white. On the left side no similar appearances were found, or, at least, there was no evident difference between the veins that remained free and those which contained coagulated blood.

In the following case also the lesions of the veins on each side were in exact proportion to the degree of infiltration.

Case VI. Lecomte, a young woman, æt. twenty-two, was admitted March 4th, in the last stage of a consumption. The only symptom that appeared remarkable in this patient was the excessively œdematous state of the right lower extremity: there was neither pain, redness, nor any lesion of the superficial veins, which could account for this symptom. Instructed by the previous cases, I predicted to my colleagues, MM. Danyau, Lemoine, Mussot, Audiat, Dumoutier, Bessière, &c. that we should find the large venous trunks obstructed by fibrinous coagula. It is proper to state that the œdema of the limb had only existed for eight days; and this fact alone was sufficient to prevent the supposition of any other lesion. The patient died on the fourth day from her admission.

The whole of the deep-seated veins in the right side, including the common and external iliacs, were filled with a coagulum, which was partly firm and black, and recently formed, especially in the secondary branches. The coagulum was traced through the principal branch, from the vena cava to the heel: all the vessels in which it existed were much thickened. On the left side there were also some sanguineous concretions in the iliac and the origin of the crural veins: they were evidently recent, and, as compared with the right side, of very small size, and did not form a continued coagulum which could obstruct the circulation of the blood.

In other and similar cases I had not been fortunate enough to detect the "point de départ" of the obstruction to the free current of blood. In this instance, however, the lumbar and mesenteric glands were tuberculated: they were as large as a nut, and several of them, agglomerated together, formed, in different parts, masses like small apples. One of these masses was situated over the inferior vena cava, where it penetrates the liver, and several others placed before it must have compressed the vein in different points when the patient was in bed, to which she had been confined for nearly ten months.

The following case will tend to confirm the conclusions that may be deduced from the above-related examples of disease, although I omitted to note the degree of infiltration of each limb, and there are some important deficiencies in the post-mortem examination.

Case VII. Marie Bissan, æt. sixty-six, died the 13th June, 1829, at the Charité, of hypertrophy of the left side of the heart and chronic peritonitis. All the abdominal viscera were agglomerated and confounded together, from the effects of the peritoneal inflammation; the omentum and mesentery were thickened; the whole formed an inextricable and heavy mass, resting upon the vertebral column. Behind this mass, which extended even into the pelvis, the aorta, vena cava, and iliac veins were pressed down. The aorta was much diminished in size, and at its bifurcation was not larger than the brachial artery of a middle-sized woman; the size of the vena cava was also equally diminished: its cavity was free. Both the iliac veins, from one end to the other, and especially the left, were filled with a red fibrinous substance, grumous on the right side, but on the left forming a continued mass; a coagulum which extended up to the crural arch, and which unfortunately was not further traced. In this case, of course, both the inferior extremities were œdematous. During life, it had been supposed that effusion had taken place in the limbs as the consequence of disease of the heart.

If we add these facts to those already recorded by Morgagni,

Hodgson, Travers, Bodson, &c., and lastly by M. Bouillaud, we may positively infer that the partial dropsy of one of the inferior extremities depends upon obstruction of the large veins which return the blood. From some of these facts I am induced to draw the same inference respecting those cases in which one limb becomes œdematous before the other, and in a much greater degree; and in this opinion I am supported by M. Louis, who in such instances has always detected fibrinous concretions, either exclusively or in a larger quantity, in the veins of the limb which was most œdematous. It may, therefore, be established as a proposition, that when one limb is infiltrated to a certain degree, and for a length of time, there is always a material obstacle to the circulation of the blood through the veins in that limb. Let it not be forgotten that passive infiltrations alone are referred to; that neither the œdema of recently delivered women, which has been explained by M. Velpeau and others in a similar manner; nor that infiltration which coexists with certain erysipelatous inflammations, or that which follows these and other exanthematous diseases, is included. The day may, perhaps, arrive when these cases may also be referred, either wholly or in part, to the theory of M. Bouillaud; but, as far as my experience extends, this would not be justifiable at present.

The obstacle which impedes the circulation of blood in the veins may be of various kinds. Thus, a tumor situated in the course of the vessels, or the impregnated uterus, may produce the same effect as sanguineous concretions formed in the vessels; but it is these concretions we most generally find. But what are we to think of those cases in which no compression is exerted on the vessels? Are we to attribute the impeded circulation and coagulation of the blood to weakness of the general economy, to an atonic state? It is certain that we find these conditions almost exclusively in weak subjects, or those exhausted by diseases. In other constitutions, the agents of the venous circulation, whatever they may be, triumph over the obstacle. Still, however, I am not disposed to adopt this explanation: it must be remarked, that the parietes of the veins, in such cases, are always hypertrophied, thicker, firmer, and more opaque than in their natural state. They nearly resemble arteries. Sometimes even the surrounding cellular tissue participates in the induration, as was found to be the fact in Case II.: this evidently supposes a state of inflammation, or chronic irritation. Should we not, therefore, attribute the coagulation of the blood to slight inflammation of the veins? This we know to be the ordinary effect of this inflammation; and the pain of which the first and second patients complained, strengthens this opinion. As I foresee many objections that may be urged to this doctrine, I suggest the idea merely as a conjecture. Whatever may be the origin of this alteration of the blood, the coagulation generally first occurs in the iliac vein, or the commencement of the crural: when once this point is obstructed, the blood coagulates gradually in all the collateral branches. An attentive perusal of the preceding cases, and the state of the blood in different points of the veins, can leave no doubt upon this subject.

The seven cases related may appear insufficient to confirm the pathological doctrines advanced in this paper, if there were not many others of a similar nature: I should, therefore, mention that the above are selected from twenty similar cases, two thirds of which I have omitted, either because they were deficient in important facts, or were merely a repetition of the others. In cases III. and IV. no particular appearances were found in the veins of one of the limbs, which was nevertheless very œdematous: here the infiltration was secondary, and of that kind which almost always takes place towards the fatal termination of chronic maladies, in consequence of a languid circulation of blood.

